# Platelet-Rich Plasma in Split-Thickness Skin Graft Donor Sites: A Narrative Review of Healing Outcomes and Pain Reduction

**DOI:** 10.7759/cureus.97921

**Published:** 2025-11-27

**Authors:** Pedro Fabian Lopez Aldana, Maria Camila Rojas Gomez, Jorge Rueda Gutierrez, Juan Darío Alviar Rueda, Laura Sofia Gutierrez, Christian Tavera Sanabria

**Affiliations:** 1 Department of Plastic and Reconstructive Surgery, Universidad Industrial de Santander, Bucaramanga, COL; 2 Faculty of Medicine, Universidad del Rosario, Bogotá, COL; 3 Department of Medicine, Universidad Industrial de Santander, Bucaramanga, COL

**Keywords:** autografts, burns, pain, plastic surgery procedures, platelet-rich plasma, postoperative period, transplant donor site, wound healing

## Abstract

Split-thickness skin autografts are the most widely used strategy for the treatment of large coverage defects secondary to trauma, burns, oncologic resections, and ulcers. However, donor site management remains a challenge, as it is associated with considerable morbidity due to pain, bleeding, pruritus, and delayed healing, often greater than in the grafted area. In this scenario, platelet-rich plasma (PRP) has gained popularity as an adjuvant therapy, with favorable results in reducing these complications. The objective of this study was to narratively review the scientific literature on the use of PRP in donor sites of split-thickness skin grafts (STSGs), with emphasis on pain control and healing outcomes. A narrative literature review was conducted, selecting key publications indexed in PubMed, Medical Literature Analysis and Retrieval System Online (MEDLINE), and Elsevier. The collected information included mechanisms of action, application techniques, clinical outcomes, and pain reduction. Additionally, institutional experience and certain relevant technical aspects are discussed. PRP accelerates epithelialization, reduces postoperative pain, decreases complications at donor sites, and improves healing quality, thereby promoting faster recovery and lower morbidity. These benefits establish it as a useful and accessible therapeutic option in this clinical setting.

## Introduction and background

Split-thickness skin grafting (STSG) is the standard surgical technique for reconstructing large skin defects resulting from trauma, burns, oncologic resections, and/or chronic ulcers. Although effective, this procedure creates a secondary wound at the donor site, which is associated with significant morbidity, including pain, bleeding, delayed healing, hypertrophic scarring, and an infection rate up to 24% [1-3]. These complications may negatively affect recovery and prolong hospitalization. Given their clinical impact, there has been growing interest in developing therapeutic strategies that optimize wound healing and minimize complications.

Among these, platelet-rich plasma (PRP) has gained increasing popularity as an adjuvant treatment in wound management [2]. PRP is an autologous concentration of platelets that releases growth factors and promotes tissue repair. PRP is obtained through centrifugation of peripheral blood, and its therapeutic potential lies in the α-granules of platelets, which contain a wide array of bioactive molecules such as transforming growth factor beta (TGF-β), platelet-derived growth factor (PDGF), vascular endothelial growth factor (VEGF), epidermal growth factor (EGF), and basic fibroblast growth factor (bFGF), among others. These mediators stimulate cellular proliferation, keratinocyte migration, angiogenesis, and modulation of inflammation, key processes in effective tissue repair [3].

PRP has been widely applied in plastic and reconstructive surgery for various indications, including acute and chronic wounds, bone regeneration, and aesthetic procedures. Its use in donor sites of STSGs may offer significant clinical benefits, such as accelerated re-epithelialization, reduced pain, and improved scarring [2,3]. However, despite promising results, the literature shows methodological heterogeneity and a lack of high-quality evidence. Previous systematic reviews assessing PRP for wound healing have consistently noted these limitations as major barriers to drawing robust evidence-based conclusions [2].

Despite the expanding use of PRP, a notable gap persists in the literature regarding a focused synthesis of evidence specifically addressing its application to donor sites of STSGs [2]. Therefore, this narrative review aims to critically evaluate and summarize the available clinical evidence on the efficacy of PRP in improving healing outcomes and reducing morbidity in these donor sites, supporting its potential role in reconstructive surgical practice.

## Review

Methodology

This review was designed as a narrative synthesis; therefore, no meta-analysis or meta-regression was performed [1]. It was conducted with the aim of synthesizing and analyzing the current literature on the use of PRP in the management and healing of donor sites in STSG. The purpose was to explore the extent to which PRP contributes to improving clinical outcomes, particularly in pain control and wound regeneration. To guide the review process, the following research question was formulated based on the Population, Intervention, Control, and Outcomes (PICO) framework: What are the observed outcomes in terms of healing and pain in donor sites treated with PRP? Within this framework, the population comprised patients undergoing STSG; the intervention consisted of PRP application to the donor site following graft harvesting; no formal comparison group was included due to the narrative nature of the review; and the main outcomes of interest were healing time (re-epithelialization) and pain at the donor site. The literature search was carried out in three major databases: PubMed, Medical Literature Analysis and Retrieval System Online (MEDLINE), and Elsevier.

The search was restricted to articles published within the last 10 years (2015-2025) in order to include the most recent and relevant evidence. This time frame was selected under the rationale that it encompasses the most significant advances and current clinical practices related to the use of PRP in wound healing, particularly in donor sites. The search strategy combined broad yet specific keywords, including “Platelet Rich Plasma,” “Split-Thickness Skin Graft,” “Wound Healing,” “Tissue Regeneration,” and “Pain,” using Boolean operators (AND, OR) to optimize sensitivity and specificity. The core search string applied was: (“platelet-rich plasma” OR “PRP”) AND (“split-thickness skin graft” OR “STSG”) AND (“wound healing” OR “epithelialization”). Minor adaptations were made for each database [2]. 

Studies were considered eligible for inclusion if they focused on human subjects who underwent STSG with PRP application at the donor site. In addition, studies were required to report clinically relevant outcomes such as pain reduction, healing time, or re-epithelialization and to be published in English or Spanish with full-text availability. Studies were excluded if they were conducted in animals or in vitro, did not address donor site management, or provided insufficient methodological or outcome data. The main variables of interest included donor site pain-evaluated through subjective scales, pain duration, or analgesic requirements-as well as healing outcomes such as time to complete re-epithelialization and wound appearance. Secondary variables included pruritus, complications (e.g., infection or delayed healing), and patient satisfaction when available.

**Figure 1:**
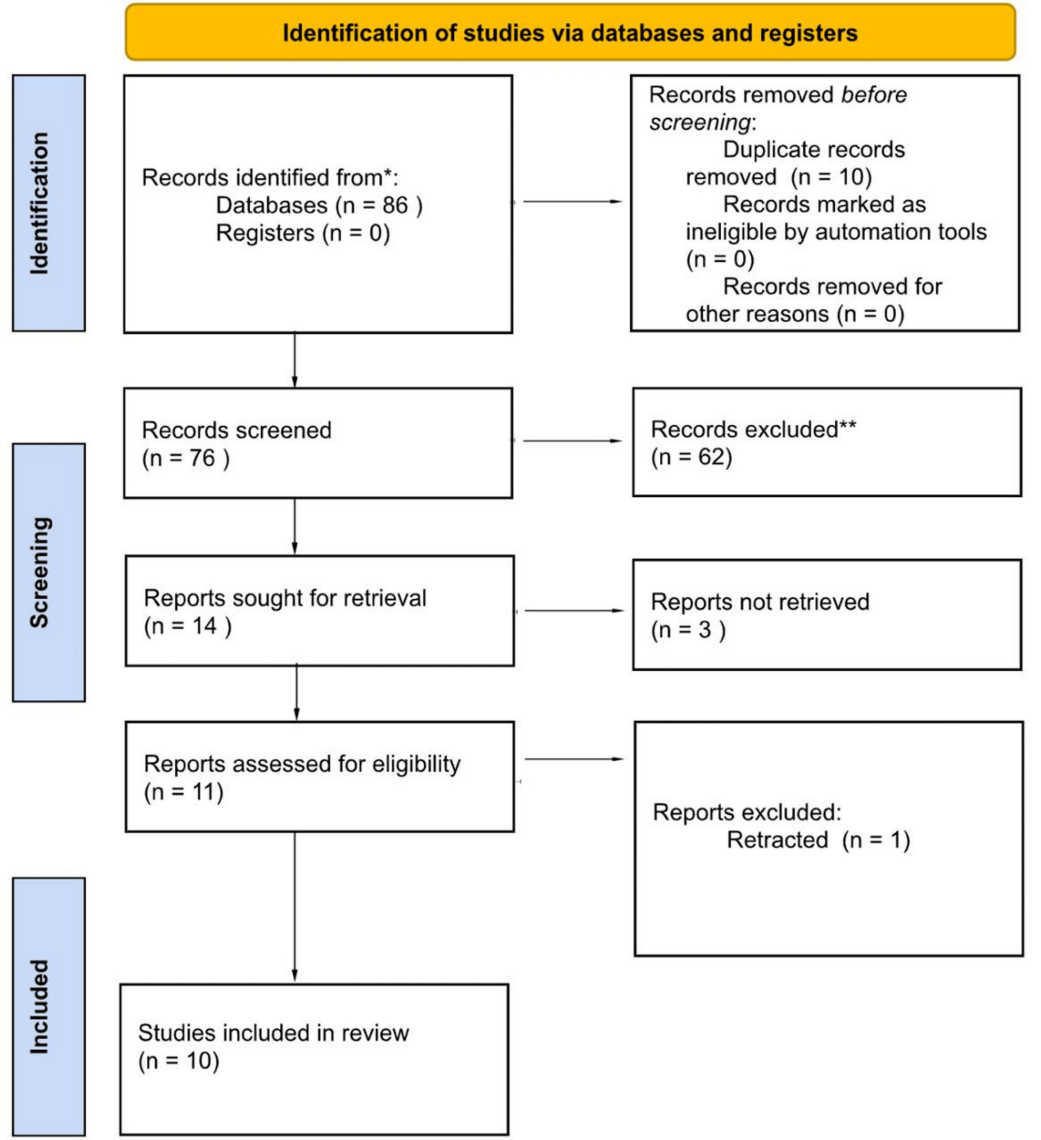
Results of the literature search and screening process

The study selection process was conducted in several stages. Initially, 86 records were identified through the database search strategy, with no additional records obtained from registers. Before screening, 10 duplicate records were removed, resulting in 76 unique records for initial screening. Titles and abstracts of these 76 records were reviewed, and 62 were excluded for not meeting the eligibility criteria. The remaining 14 reports were sought for full-text retrieval, but three could not be accessed. A total of 11 reports were then assessed for eligibility, of which one was excluded because it had been retracted. Ultimately, 10 studies met all inclusion criteria and were incorporated into the final narrative review (Figure 1). Data selection and extraction were performed independently by two reviewers. Any discrepancies or disagreements during the process were resolved through discussion and, when necessary, with the involvement of two academic supervisors. All references and extracted data were systematically organized and managed using Mendeley software (Elsevier, Amsterdam, Netherlands) to ensure proper traceability and consistency throughout the review process. No formal risk-of-bias tool was applied due to the narrative design of this review. Nonetheless, the included studies present several methodological limitations that may introduce bias, such as small sample sizes, heterogeneity in PRP preparation and application protocols, and inconsistent reporting of healing outcomes. These factors should be considered when interpreting the findings [3]. 

Review

Overview of the Evidence

The collected evidence consistently indicates that the use of PRP in donor sites of STSGs leads to faster re-epithelialization, reduced early postoperative pain, and improved overall scar quality when compared with conventional management. These benefits are documented across randomized trials, intrapatient comparative studies, and observational cohorts, which describe earlier epithelial coverage within the first week, faster transition to complete epithelialization by weeks two to three, lower analgesic requirements, and more favorable scar evolution during follow-up [2-9].

In addition, several studies report a reduction in infectious complications in PRP-treated donor sites, contributing to smoother recovery [4,8]. Evidence from burn populations mirrors these findings, demonstrating shorter re-epithelialization times, decreased pain, lower treatment costs, and improved outcomes in standardized scar quality scales when PRP is used as an adjuvant therapy [10,11].

Biological Rationale and Mechanisms

The effect of PRP is not uniform across all clinical scenarios. Factors such as the timing of application, the patient's systemic condition, and the method of preparation/application of the concentrate influence the magnitude of the outcomes. In studies involving burn patients, particularly those with deep burns, the benefits of PRP are more evident during the early phases of wound healing, whereas differences in later stages or long-term follow-up tend to diminish [12]. Nevertheless, from a biological perspective, PRP offers clear advantages. Experimental models and translational studies have shown that it generates a microenvironment rich in growth factors and cytokines that stimulate new blood vessel formation, promote keratinocyte migration, and modulate the inflammatory response. Furthermore, its effectiveness may be enhanced through sustained-release delivery systems by combining it with mononuclear cells, which experimentally has translated into greater neovascularization and faster epithelization [3,13-14]. In contrast, donor sites managed with conventional care exhibit variable morbidity, with prolonged closure times, significant pain during the first week, and complications related to the type of dressing. In this context, interventions that accelerate epithelization and reduce pain, such as PRP, provide a clear clinical advantage [2]. Moreover, in resource-limited settings, the autologous nature and relatively low cost of PRP, compared with advanced dressings or more complex technologies, make it a practical and accessible alternative, with the potential to improve not only the speed of healing and pain but also the final quality of the scar [15,16]. A summary of the studies is provided in Table 1.

**Table 1:** Overview of published studies evaluating PRP in donor site healing PRP: platelet-rich plasma; PRGF: plasma rich in growth factors; VAS: Visual Analog Scale; TBSA: total body surface area; POSAS: Patient and Observer Scar Assessment Scale

Main author	Type of study	Number of patients	Patient age (mean)	Sex		Indication	Compared intervention	Need for analgesics	Pain	Healing	Dressing change frequency (days)	Surgical site infection	Hospital stay (days)	Follow-up time (days)
				Female (%)	Male (%)									
Slaninka I, et al., 2020	Randomized clinical trial	24	66.5 years (range: 18–91 years)	15 (62.5%)	9 (37.5%)	Chronic non-healing leg wounds, trauma, burns	Grafts with PRP + Vaseline gauze vs. grafts with Vaseline gauze	Not evaluated	Not evaluated	With PRP: 14.9 days, Without PRP: 18.4 days, difference: average reduction of 17.8% (~3.5 days faster)	Not evaluated	Not evaluated	Not evaluated	9–40 days
Dhua S, et al., 2019	Randomized clinical trial	40	35.5 years (range: not reported)	16 (40%)	24 (60%)	Trauma, chronic ulcers, burns, and infections	Grafts with PRP vs. grafts without PRP	Not evaluated	Patients with PRP grafts: less postoperative pain, itching, and irritation	Patients with PRP grafts: faster healing, with less edema, hematoma, and graft loss	PRP grafts: 2.75. non-PRP grafts: 8.15	Not evaluated	PRP grafts: 15.2. non-PRP grafts: 17.3	90 days
Ali SS, et al., 2022	Prospective study	15	37.47 years (range: 24–54 years)	5 (33.3%)	10 (66.6%)	Burns, trauma, tumor resection area	Grafts with PRP + paraffin gauze on the proximal graft area vs. grafts with paraffin gauze on the distal graft area	Not evaluated	Day 7: PRP 1.73/10, non-PRP 4.67/10, Day 14: PRP 1/10, non-PRP 2.73/10, Day 21: PRP 1/10, non-PRP 1.4/10	Day 7: PRP 44.53% not epithelialized, non-PRP 68.47% not epithelialized; Day 14: PRP 16% not epithelialized, non-PRP 30.4% not epithelialized; Day 21: PRP 0.2% not epithelialized, non-PRP 0.67% not epithelialized	Days 7, 14, and 21	Not evaluated	Seven days hospitalized after graft harvesting	21 days
Jain RK, et al., 2021	Prospective randomized clinical study	20	18–80 years (mean not reported)	8 (40%)	12 (60%)	Not specified	Grafts with PRP + Vaseline gauze on the proximal graft area vs. grafts with Vaseline gauze on the distal graft area	Not evaluated	Day 7: PRP 6.5/10, non-PRP 8.4/10; Day 14: PRP 3.5/10, non-PRP 6.35/10; Day 21: PRP 2/10, non-PRP 4.2/10	Accelerated healing was observed in wounds treated with PRP compared to those without PRP on days 7, 14, and 21.	Days 7, 14, and 21	Not evaluated	Not evaluated	21 days
Fang Z, et al., 2019	Retrospective study	30	PRP group: mean age 37 years; non-PRP group: mean age 35 years (range: 19–62 years)	13 (43.3%)	17 (56.6%)	Burns, scar deformities, and acute and chronic wounds	IGrafts with PRP covered with Vaseline gauze vs. grafts with Vaseline gauze	Not evaluated	PRP showed no difference in pain on Day 3, but a significant reduction between Days 7 and 14, which disappeared by Day 21.	Mean wound healing time was 13.89 ± 4.65 days in the PRP-treated group and 17.73 ± 5.06 days in the Vaseline gauze group (p < 0.05).	Not evaluated	Not evaluated	Not evaluated	Scar follow-up (Vancouver Scar Scale) up to 364 days. Pain was also evaluated with VAS up to 21 days.
Marck RE, et al., 2016	Randomized, double-blind, controlled trial	52	51.2 years (range: 20–90 years)	21 (40%)	31 (60%)	Deep and full-thickness burns ≥ 2% TBSA	Grafts with PRP vs. grafts without PRP	Not evaluated	No statistically significant difference in mean pain scores between areas with and without PRP.	When categorized, areas treated with PRP more frequently showed “equal or better” graft integration and epithelialization. Among patients operated ≤ 7 days after burn injury, PRP provided a significant benefit.	Initial dressing removal at five to seven days; then twice-weekly follow-ups until complete healing	No significant differences in bacterial colonization rates were found between PRP and control areas	Not evaluated	Short term: main evaluation on days 5–7; pain measured during the first week; cultures until wound closure. Long-term (scar): follow-up at three, six, and 12 months postoperatively.
Chigurupati VS, et al., 2023	Non-randomized controlled clinical trial	60	<40 years = 32 (53.3%); >40 years = 28 (46.7%)	21 (35%)	39 (65%)	Trauma, burns, oncologic resection	Grafts with PRP vs. grafts without PRP	PRP grafts: no postoperative opioids required. Non-PRP grafts: four patients required opioids.	PRP grafts: less pain during the first five postoperative days; Non-PRP grafts: more pain during the first five days; four patients required opioids.	>50% healing: 86.7% PRP vs. 53.3% control	<2 dressing changes: 86.7% PRP grafts vs. 13.3% non-PRP grafts	PRP grafts: 3.3%; non-PRP grafts: 23.3%	<10 days: 90% in PRP grafts vs. 36.7% in non-PRP grafts >10 days: 10% in PRP grafts vs. 63.3% in non-PRP grafts	90 days
Orlandi C, et al., 2018	Preliminary observations of a case series	2	72 years (range: 63–81 years)	2 (100%)	0 (0%)	Refractory plantar ulcers due to erosive lichen planus + extensive lesions from Darier’s disease	Grafts with PRP (no control group)	Not evaluated	Rapid clinical reduction of pain reported after the procedure (qualitative data).	Complete clinical healing in approximately three weeks in both cases.	Not evaluated	Not evaluated	Not evaluated	Initial follow-up: healing evaluated at three weeks ≈ 21 days. Long-term follow-up: Case 1 = 4 years ≈ 1460 days; Case 2 = 1 year ≈ 365 days.
García-Sánchez JM et al., 2022	Randomized clinical trial	20	40 years (range: 28–51 years)	5 (25%)	15 (75%)	Second- and third-degree burns	Grafts with PRP + hydrocolloid vs. grafts with PRGF vs. hydrocolloid grafts with hydrocolloid	Not evaluated	PRP and PRGF grafts showed less pain in the first five to eight days, but the difference was not statistically significant.	PRP: 8 days; PRGF: 11 days; hydrocolloid: 11 days; PRP and PRGF achieved better scores on the Vancouver and POSAS scar assessment scales.	Not evaluated	Not evaluated	Not evaluated	180 days
Gupta S, et al.,2020	Randomized clinical trial	200	Case group: mean age 45.1 years; Control group: mean age 48.2 years	93	107	Burns and ulcers in the healing phase	Grafts with PRP + paraffin gauze vs. grafts with paraffin gauze	Not evaluated	PRP reduced pain (qualitative emphasis).	PRP increased initial graft adherence (Day 2) with statistical significance (p = 0.04).	First dressing change on Day 2, then every other day	In the control group, six patients developed signs of local infection with partial graft loss. In the PRP group, two patients experienced approximately 50% graft loss.	Not evaluated	6 days

Clinical Integration and Evidence Synthesis

The critical integration of findings suggests that PRP is a useful, safe, and clinically relevant tool to optimize the healing of donor sites in STSGs by accelerating re-epithelialization, reducing postoperative pain, and, in several studies, improving scar quality, as well as lowering the incidence of infectious events [2-10]. The agreement between systematic reviews, meta-analyses, and primary studies further reinforces the reliability of PRP’s clinical impact. Intrapatient methodologies, in particular, minimize interindividual variability and strengthen the internal validity of the evidence base [3,5,12].

Sources of Heterogeneity and Limitations

Nevertheless, sources of heterogeneity remain that must be considered before recommending indiscriminate adoption. These include (i) differences in preparation (platelet concentration, activation, gel versus liquid), (ii) variation in dose/volume and frequency of application, (iii) combination with different types of dressings, and (iv) nonuniform evaluation time points. These variables may account for the wide range of reported effects and the attenuation of differences in certain scenarios (e.g., deep burns with delayed application), without invalidating the overall favorable trend of PRP. Therefore, the standardization of protocols, including preparation, activation, volume per area, synchronization with graft harvesting, and dressing regimens, remains a priority for future research [2,4,10-12].

Clinical Implications

From a clinical perspective, PRP may be incorporated when the therapeutic goal is to enhance wound healing and optimize postoperative care. Its autologous nature, ease of preparation, and relatively low cost make it a practical adjunct when adequate institutional protocols exist. Implementation should include standardized preparation parameters, documentation of application timing and concentration, and the use of validated tools to measure outcomes such as epithelization time, pain, complications, and scar quality. These practices support protocol development, reproducibility, and a broader clinical adoption [3-16].

Future Directions

To consolidate the evidence and facilitate its incorporation into clinical practice, multicenter randomized clinical trials are required, with adequate sample sizes, standardized outcome measures, and follow-up periods that include both aesthetic and functional endpoints in the medium and long term, as well as cost-effectiveness analyses tailored to each healthcare system [2,4,10,12,15-16]. In conclusion, PRP represents a valuable adjuvant for donor sites of STSGs. The robustness of the clinical evidence, its biological rationale, and the feasibility of its implementation support its role as a safe and effective option. Its benefits are most evident when preparation is optimized and when applied in contexts where rapid wound closure and patient comfort are prioritized [2-16]. 

## Conclusions

PRP has emerged as a promising alternative in the management of STSG donor sites, offering significant improvements in wound healing as well as reductions in pain and local complications. Its high content of growth factors stimulates tissue regeneration and accelerates the re-epithelialization process, which not only decreases patient morbidity but also contributes to a faster and more satisfactory recovery. These benefits reinforce the importance of incorporating PRP as part of therapeutic strategies in this clinical setting.
